# Assessment of the water sources for potential channels of faecal contamination within Vhembe District Municipality using sanitary inspections and hydrogen sulphide test

**DOI:** 10.1038/s41598-023-33551-y

**Published:** 2023-04-17

**Authors:** A. Murei, I. Kamika, A. Samie, M. N. B. Momba

**Affiliations:** 1 Department of Environmental, Water and Earth Sciences, Tshwane University of Technology, Arcadia Campus, P/B X 680, Pretoria, 0001 South Africa; 2 Nanotechnology and Water Sustainability Research Unit, School of Science, College of Science, Engineering and Technology, Florida Campus, University of South Africa, P.O Box 392, Florida, 1710 Roodepoort South Africa; 3 Molecular Parasitology and Opportunistic Infections Research Program, Department of Biochemistry and Microbiology, University of Venda, Private Bag x5050, Thohoyandou, 0950 South Africa

**Keywords:** Environmental impact, Natural hazards, Microbiology techniques, Risk factors, Bacteria, Environmental microbiology

## Abstract

Numerous human activities and poor sanitation management cause public health concern, particularly in rural communities without reliable water supply systems and resources for the monitoring of the quality of their water sources. This study assessed the relationship between observed sanitary risks and hydrogen sulphide (H_2_S) strip test results in the identification of faecal contamination of various water sources used at household level in rural areas of the Vhembe District Municipality. The highest percentage sanitary risk scores ranging from 50 to 100% were recorded for both river and dam water commonly used by the households for multiple purposes, including drinking. All the surface water samples (100%) also tested positive for H_2_S production, which is linked to the contamination of water sources by bacteria of faecal origin. The overall results showed a significant and positive correlation (r = 0.623, p = 0.003 in the wet season and r = 0.504, p = 0.017 in the dry season) between sanitary risk scores and H_2_S strip test results. In low resource settings, the use of sanitary inspections combined with the inexpensive and easy-to-use H_2_S strip tests can be effective as drinking water quality management tools to raise an awareness among community members of the faecal contamination of their water sources.

## Introduction

The demand for reliable water sources for domestic use is growing, especially among the poor in the majority of developing countries, and South Africa is no exception. It is estimated that 785 million people worldwide still use unimproved drinking water sources^1^, which include unprotected wells and springs and surface water. Most of the people depending on unimproved drinking water sources live in developing regions of sub-Saharan Africa and Southern Asia^2^. Water utilities struggle to sustainably deliver clean water to millions of people, particularly in rural communities, although the level of faecal contamination in the water is low due to close monitoring of water sources^3–5^.

In addition to the lack of access to clean water sources, rural communities also lack access to improved sanitation. A report by the World Health Organization (WHO) revealed that 673 million people still practise open defaecation, and 2 billion people lack access to proper sanitation worldwide^6^. Open defaecation is practised in the fields, bushes and bodies of water or other open spaces. These ‘faecal fields’ potentially pose health problems to rural communities and place water sources at risk of flooding with faecal material from surrounding areas during heavy rains^7^. Unsanitary practices such as defaecation in stream channels and riverbeds during dry seasons have been reported to contribute to faecal contamination on boundaries of water bodies^8^. However, unimproved water sources have been found to harbour higher rates of faecal contamination, which is one of the main causes of waterborne diseases in households for those who use these water sources for drinking purposes. For example, in 2021, 23 countries reported cholera outbreaks, mainly in the WHO Regions of Africa and the Eastern Mediterranean and this trend continued into 2022 with over 29 countries reporting cholera cases or outbreaks. Sub-Saharan Africa remains the epicentre of cholera outbreaks from 1989 to 2022^8^. Acute diarrhoeal outbreaks have been reported in Pakistan and Sudan^9,10^. In 2016, diarrhoeal diseases brought on by poor access to water, sanitation, and hygiene resulted in 1.6 million fatalities and 105 million DALYs (disability-adjusted life years) in low- and middle-income countries^11^. Most diarrhoeal deaths can be avoided through adequate management of water sources, sanitation, and better hygiene practices.

In South Africa, pollution of rivers is well-known. Nevertheless, most river health studies and programmes have mainly concentrated on evaluating the river water quality of large rivers, like the Great Letaba, Limpopo, Crocodile, Olifants, Thukela, Orange, Vaal, and Inkomati. The majority of the smaller rivers that feed into these larger rivers have been overlooked or forgotten^12^. Monyai et al.^12^ evaluated the water quality of tributaries of the Luvuvhu River in the Limpopo Province, South Africa (Thulamela Local Municipality, Vhembe District), including Dzindi, Mutshindudi, Mvudi, and Lukunde, and results showed a high level of total acidity in some water sources indicating water pollution^12^. Diffuse or non-point source pollution remains the significant barrier to meeting good water quality standards, especially in rural communities with limited resources^13^. Furthermore, unlike point source pollution, which arises from a single source (for example sewage or industrial effluent discharge points), non-point source pollution does not come from a single source and is difficult to manage^14^. Faecal pollution of water sources in rural communities is mainly caused by non-point source pollution such as human faeces and animal droppings, agriculture pollutants, poor sanitation management, etc^15,16^. Therefore, using a sanitary inspection (SI) to identify potential sources of faecal pollution and pathways of pollution may assist in the management of high-priority risk concerns, thereby protecting public health.

In rural communities, human and animal waste are the common sources of surface water and groundwater pollution^17^. Domestic wastewater from on-site wastewater disposal systems (such as septic tanks and pit-latrines) contains a number of enteric pathogens that could pose a health risks to groundwater. In addition, the excreta of humans and warm-blooded animals could potentially be utilised as fertilisers in agriculture since they contain plant nutrients^18^. In some cases, heavy rainfall events can have a significant impact on microbial water quality due to runoff that carries faecal matter into surface water sources, and water contaminated by enteric pathogens (especially water from unprotected sources) may pose risks of waterborne diseases^19^.

Microbial water quality must be monitored regularly. In a case where microbial contamination is detected, water should be subjected to minimum treatment such as boiling and addition of bleach prior to human consumption. Rural water sources are the least monitored, yet they have the highest levels of faecal contamination. Monitoring for faecal contamination of drinking water in rural areas is limited by the lack of laboratory resources, funding and skilled personnel^20^. Creating an awareness of water contamination and the risks involved and thus the need for regular monitoring of microbial quality and minimum treatment of water can be accomplished, even in rural communities with limited resources. There is, therefore, a need for the use of an affordable test kit for field tests of water quality in rural communities.

Hydrogen sulphide (H_2_S) detection tests, which are cost-effective can be used to evaluate whether bacteria of faecal origin are present in the water^5^. These bacteria can reduce organic sulphur to sulphide as H_2_S gas. This test kit method relies on the detection of faecal coliform bacteria that produce hydrogen sulphide rather than non-faecal coliform bacteria. These faecal coliform bacteria are present in the intestines and faeces of warm-blooded animals. It is a method for examining the microbiological quality of drinking water on-site^21^. The H_2_S strip test works as a presence/absence test; the solution will change colour to black in the presence of H_2_S producing organisms. With the goal of enhancing the quality of drinking water and reducing the burden of diseases associated with water, the H_2_S strip test is an effective method that enables the users to determine whether their water source is fit for consumption^22^. For more than 30 years, H_2_S strip tests have been used successfully to identify faecal contamination in water around the world^23^. The H_2_S tests are nonetheless comparable to thermotolerant coliform tests while being able to detect a considerably wider variety of microorganisms; an average sensitivity (CI95 80–92%) and specificity (CI95 72–90%) of 87% and 82%, respectively, have been reported^23^. Hence, culture-based methods and molecular microbiology can be used to confirm the H_2_S assays for bacterial genera related to faecal contamination.

For environments with limited resources, the H_2_S strip test is a particularly effective water quality monitoring instrument^24^. The government of India has gradually approved the tests for use in community water points for initial monitoring, with positive results requiring laboratory-based testing to confirm them. However, the WHO^2^ Guidelines for Drinking Water do not currently suggest the H_2_S test. In spite of this, the H_2_S strip test has become an essential component of any household-based rural water quality surveillance programme. This technique has been promoted by UNICEF and is frequently used as a presence/absence test in developing nations and outlying regions^25^. The H_2_S test kit is easy to use and affordable for the average family. Manja et al.^21^ proposed the hydrogen sulphide (H_2_S) method as a low-cost field test to identify faecal pollution of water in such settings. The approach of empowering communities by equipping them with the above-mentioned simple tools and training local facilitators is seen to be successful and has the potential to be replicated in rural communities. However, the role and efficacy of H_2_S tests for sanitary risk assessment and water quality testing at the level of rural communities have not yet been investigated in diverse water sources used in rural communities with intermittent or no water supply.

Methods such as modified sanitary inspections and the hydrogen sulphide test may be used in rural regions because they need to be aware of any possible contamination risk. Therefore, the goal of this study was to assess the relationship between observed sanitary risks and the hydrogen sulphide strip test results in the identification of faecal contamination in various water sources. These two methods may be used as drinking water quality management tools to raise an awareness among rural community members of the faecal contamination of their water sources. The following four objectives were pursued to achieve the main goal of the study: surveying; using sanitary inspections (SIs) to assess the risk of microbial contamination; evaluating the effectiveness of the use of H_2_S paper strips in the research area; and establishing a connection between the sanitary inspection and the H_2_S strip test for microbial risk categorisation. In order to reduce the disease burden, this study also serves as a foundation for future extensive research on water, sanitation, and hygiene in this region.

## Results

### Demographic information of the study areas

The results in Table 1 already published by Murei et al.^26^ were considered to highlight the level of education, the employment rate and the predominant waterborne diseases in the study areas. Briefly, most of the participants attained either primary, secondary, or tertiary level education and very few did not go to school at all. The overall survey showed that almost 70.5% of the residents of the Vhembe District Municipality (VDM) are employed. Of all the participants, 15.1% reported that they experience diarrhoeal disease, with 40% of them indicating the occurrence of frequent episodes of diarrhoea.

**Table 1:** Overall demographic information of the Vhembe District Municipality.

Characteristics	Number	Frequency (%)
Educational level		
No schooling	66	2.3
Schooling	2815	97.7
	N = 2881	
Employment		
Unemployed	568	29.5
Employed	1359	70.5
	N = 1927	
Diarrhoea occurrence	209	15.1
	N = 1388	
Diarrhoea frequently occurring	85	40.7
	N = 209	

### Water sources used in the Vhembe District Municipality

As can be seen in Table 2, most of the households in the rural communities under this study used piped water supplied by the municipality as their main water supply and only about 8.4% used alternative water sources. However, people frequently turn to alternative water sources and this is due to the fact that the water supply is inconsistent. Overall, most people rely on rainwater (n = 333, 47.1%) and boreholes (n = 123, 17.4%) for drinking, irrigation, and other domestic purposes. Other alternative water sources that are used includes springs 6.9%, dams 1.6%, hand-dug wells 1.3%, and rivers 7.5% with females being the ones who mainly fetch water for households. One hundred and forty-six respondents indicated that they treat this water source before drinking mostly using household treatment methods such as boiling (37%) and bleaching (26.0%). The water is used for agricultural (irrigating crops) and domestic purposes, which include drinking, cooking, washing clothes, house cleaning and bathing. Maize production and other seasonal crops made up most agricultural practices. Cattle (71.2%), donkeys (11.7%), goats (7.6%), and dogs (5.3%) are among the animals seen in the area near water sources.

**Table 2:** Water sources and their usage in the study area.

Characteristics	Number	Frequency (%)
Main water supply		
Tap water (treated)	1 272	91.6
Others (untreated)	116	8.4
	N = 1 388	
Alternative water source		
Boreholes	123	17.4
Springs	49	6.9
Dams	11	1.6
Hand-dug wells	9	1.3
Rivers	53	7.5
Stormwater	333	47.1
Others	129	18.2
	N = 707	
Animals around water source		
Cattles	280	71.2
Donkeys	46	11.7
Goats	30	7.6
Dogs	21	5.3
Others	16	4.1
	N = 393	
Agricultural activity		
Maize	21	41.2
Beetroot	2	3.9
Crops	4	7.8
Spinach	14	27.5
Onion	5	9.8
Tomatoes	5	9.8
	N = 51	
Who fetches water?		
Male	260	21.7
Female	938	78.3
	N = 1198	
Purpose of the water		
Domestic use	489	69.2
Agricultural use	218	30.8
	N = 707	
Households using water treatment methods		
Bleaching	38	26.0
Chlorination	19	13
Boiling	54	37
Salting	12	8.2
Other	23	15.8
	N = 146	

### Sanitation-related status in rural communities

Table 3 depicts the sanitation-related status in target villages under the present study. Almost every household in the Vhembe District Municipality has a toilet with 90.9% having pit-latrines and 3.8% having flush toilets connected to a septic tank. Some (2.3%) respondents stated that they still practise open defaecation due to a lack of access to toilets in their yards. About 17% of the respondents indicated that they dispose of soiled diapers in refuse bags with solid waste that are collected by the municipality, 9.5% inside the toilets, and 14.7% in open pits. For 29.7% of the households studied, the calculated distance between septic tanks/toilets and the water source was greater than 50 m, while 70.3% of households were found to have the toilet/septic tank near the water source. The soil type in the study area was found to be mainly loamy (76.2%) and only 23.3% was very fine sand.

**Table 3:** Sanitation-related status in the study area.

Characteristics	Number	Frequency (%)
Sanitation facility		
Pit-latrine	1262	90.9
Flush septic	53	3.8
Open defaecation	32	2.3
Other	41	3
	N = 1388	
Distance between pit-latrine/septic tank and water source		
≥ 50 m	412	29.7
< 50 m	976	70.3
	N = 1388	
Diaper disposal		
Buried	45	13.2
Solid waste disposal	109	32.1
Dispose of in toilets	61	17.9
Near river	5	1.5
Open pit	94	27.6
Bush	24	7.1
Burn	2	0.6
	N = 340	
Soil type		
Loamy	1057	76.2
Very fine sand	324	23.3
Other	7	0.5
	N = 1388	

### Sanitary inspection

**Figure 1:**
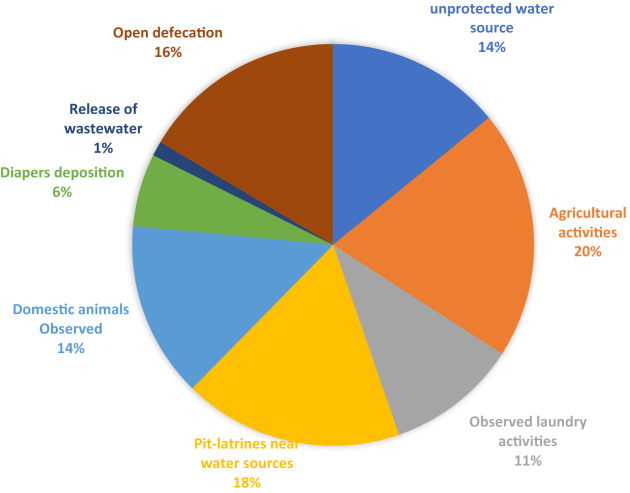
A pie chart showing observed activities near water sources in the Vhembe District Municipality.

The potential for pollution of water sources and the degree of danger are determined by human activities near these water sources. Figure 1 illustrates the various human and animal activities that cause water contamination in the VDM. Agriculture accounted for 20% of all observed activities, followed by the presence of pit latrines (18%) and evidence of open defaecation (16%), which were the most frequently encountered activities close to water sources. The Thohoyandou Wastewater Treatment Plant discharges its effluent into the Mvudi River. In half (4/8) of the surface area documented in the research region, diaper disposal sites were seen close to the water sources. With the exception of boreholes and protected springs, domestic animals were detected practically everywhere in areas surrounding water sources.

**Figure 2:**
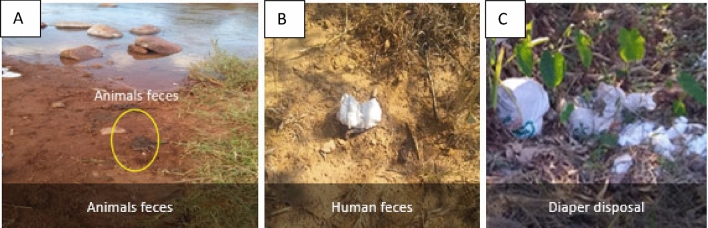
Human activities that contribute to contaminating water sources in the area. (**A**) Animal faeces located near the river; (**B**) open defaecation in the bush; (**C**) disposable diaper waste in the valley near the dam.

Sources of faecal pollution have been identified in rural communities of the Vhembe District Municipality. The data captured included faecal matter (e.g. from humans or warm-blooded animals) around water bodies, animals grazing, agricultural activities, and illegal dumping sites are also shown, depicting poor waste management as can be seen in Fig. 2.

The percentage sanitary risk scores and risk rating (Table 4) were determined according to the World Health Organization^6^ rating for water sources. The Luvuvhu River was identified as having the highest sanitary risk score, at 100%, followed by Nandoni Dam and Tshivhulani Spring, both of which had a sanitary risk score of 87.5%. These high sanitary risk scores are a cause for concern as these water sources are used by community members for domestic purposes. The only water source with the lowest risk score was Tshakhuma Spring (12.5%).

**Table 4:**
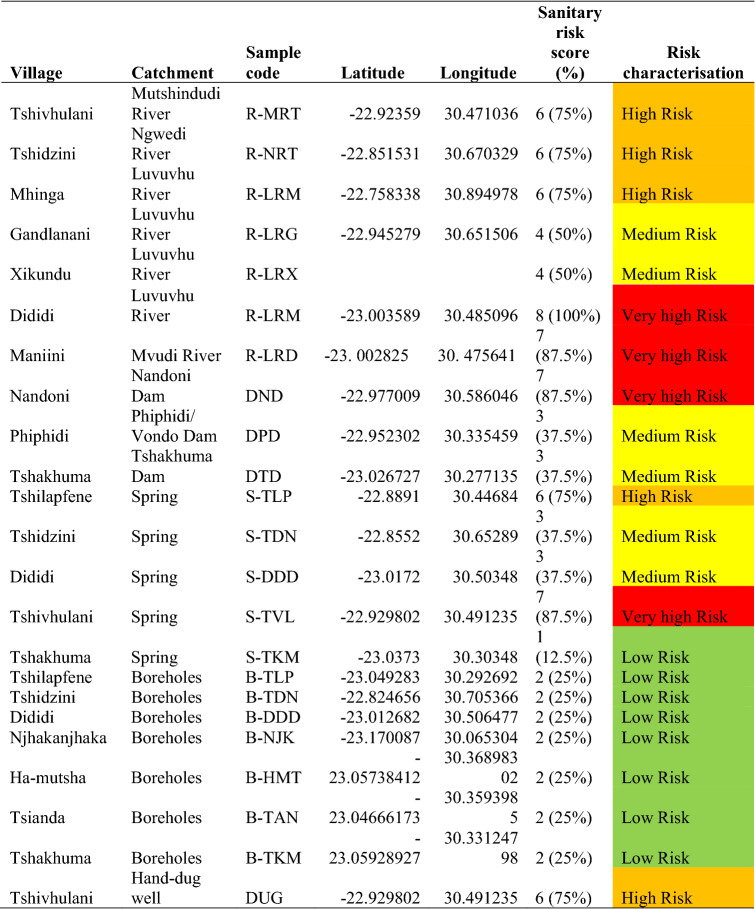
Percentage sanitary risk score and risk rating at various catchment areas located in villages in the Vhembe District Municipality.

### Water quality analysis using hydrogen sulphide test

**Figure 3:**
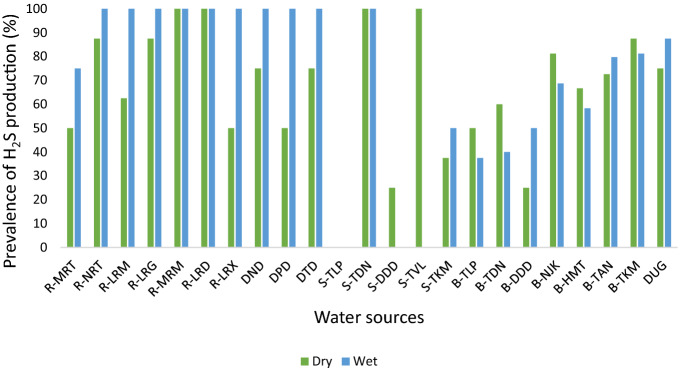
Prevalence of H_2_S production in water samples from various water sources in dry and wet seasons.

The study revealed that during the wet season, almost all the surface water samples from rivers and dams were found to be positive for H_2_S production, with the exception of the Mutshindudi River, where 75% of water samples tested positive (Fig. 3). No H_2_S gas producing bacteria of faecal origin were found in the water samples of the springs in Tshilapfene (during both wet and dry seasons), Tshivhulani, and Dididi (during the wet season). The water samples of only two springs were found to have H_2_S gas producing bacteria of faecal origin: 100% of the Tshidzini Spring water samples tested positive for H_2_S production for both dry and wet seasons, while 100% of Tshivhulani Spring water samples tested positive for H_2_S production during the dry season. None of the borehole water samples were found to test positive for H_2_S production in both wet and dry seasons.

### Correlation between sanitary risk score and H_2_S strip test results

The overall results showed a significant and strong positive correlation (r = 0.623, p = 0.003 in the wet season and r = 0.504, p = 0.017 in the dry season) between sanitary risk score and H_2_S strip test results.

## Discussion

Public health concerns are brought on by unsafe water, poor sanitation and hygiene, and some serious health conditions may even be fatal^27^. According to the Constitution of the Republic of South Africa, 1996, everyone has the right to safe drinking water and proper sanitation^28^. However, this is a luxury in rural communities and a scarce resource in peri-urban areas and townships. Rural communities are isolated, making it challenging for national surveillance agencies to regularly visit and provide advice on concerns relating to the safety of drinking water^20^. Water and sanitation in rural areas are less monitored, and the contamination of drinking water is due to non-point pollution sources, and these are difficult to manage. Therefore, community members must find a way of managing their water sources and equip themselves with water quality monitoring tools.

Understanding the main causes of water contamination and how to mitigate them depends to a large extent on education. According to the demographic data from the research region, 97.7% of people attended school, and just a small percentage did not. Therefore, community members will be able to monitor their water quality and take appropriate action as needed with the help of workshops and training for basic household water quality monitoring tools like H_**2**_S and sanitary inspections. Our findings revealed that 70.5% of people are employed, meaning that if they prioritise their water safety, they will be able to purchase cheap H_2_S test paper strips. According to current figures, environmental factors account for 94% of the burden of diarrhoeal illnesses, with contaminated water, inadequate sanitation and poor hygiene serving as their primary causes^29^. This H_2_S strip test could be very helpful in our study area since 40% of the people surveyed who experience diarrhoea indicated that it occurs frequently.

In the present study, it was noted that every village in the VDM has a water scheme designated to it that supplies households with treated potable water; however, the water supply is not reliable. This was confirmed by Murei et al.^26^ who pointed out that most of the water pipes in VDM may be dry for weeks, months and even years. Therefore, residents end up depending on untreated or contaminated surface water and groundwater. In this research area, it was found that various alternative water sources such as rainwater, rivers, dams, hand-dug wells, springs and boreholes are used. Unfortunately, various domestic animals such as cattle, donkeys, goats and dogs were found near water sources. Animal waste contamination of recreational waters, on the other hand, provides a risk to human health since waterborne disease agents such as *Campylobacte*r spp., *Cryptosporidium parvum*, and *E. coli* O157:H7 can be transmitted from animals to humans^30^. People who use the water from these rivers for household purposes may be at risk of health problems if nothing is done. This means that sanitary practices should consider local knowledge of probable animal disease sources and environmental pathways to people.

The present findings also revealed that mainly females (78.3%) are responsible for collecting water from the water sources, and some of them claimed to purify both surface water and underground water before using it for drinking purposes. Therefore, women should be given priority during H_2_S and sanitary inspection workshops and training, since they are the primary caregivers in the households. This will help in reducing waterborne diseases across such communities. None of the respondents mentioned using ceramic filters to treat their water while the ceramic pots are produced by rural communities in the study areas; the majority either boil their water or they use bleach. Therefore, thorough knowledge of household water treatment techniques should also be shared with community members to give them the opportunity to select the appropriate and most affordable option.

Agricultural activities are common sources of freshwater contamination^31^. Many people in the study areas are subsistence farmers having small gardens in their yards, and some use animal waste as fertilisers. The majority of these activities were found close to water sources. Cattle regularly produce manure (faecal matter) on the banks of rivers, which contaminates the water supplies. This manure may enter water bodies during periods of heavy rain, which could cause eutrophication, which makes the water unfit for human use and for aquatic life. A study of the Nandoni Dam on the Luvuvhu River found that eutrophication remained a threat to the water quality of this dam^32^. Hence, action must be taken in order to prevent and reduce the incidence of eutrophication.

Human actions such as inadequate sanitation management can potentially contaminate underground water. Pit-latrines and flush toilets are the on-site sanitation systems that are most commonly utilised in the research region; 90.9% of homes use pit-latrines because of insufficient water supply in the area. Additionally, the bulk of people who flush the toilet use groundwater from backyard private boreholes. Depending on the kind of soil and water table level, latrines and septic tanks are frequently connected to a soak pit (or soakaway), allowing contaminants to leach directly into groundwater sources^22^. About 70.3% of pit-latrines in the study area are near water sources. The presence of toilets in close proximity to water sources poses a high risk of faecal contamination as microorganisms can migrate from the latrines to the drinking water source^29,33^. The local government should also emphasise the enforcement that specifies the minimum distance between the water source and the toilet depending on soil characteristics of the residential stand and the depth of the water table.

In our study area, open defaecation is practised (2.3% of the respondents of our survey stated that they still regularly defaecate in the open) and human and animal excreta have been observed close to water sources^34,35^. Another problem is the disposal of soiled baby diapers close to shrubs or water sources. These activities pose a health risk to the general public as they create risks to water sources during hazardous events like heavy rainfall events causing floods, surface runoff, and seepage. One of the areas in South Africa where flooding has caused significant destruction, including the loss of life, property, and infrastructure, is the Luvuvhu River^36^. Hence, sanitary inspections, adequate sanitation management, and education of community members should be done in order to minimise this risk.

Based on the overall findings of the sanitary inspection, agricultural operations (20%) were the activities most frequently seen close to water sources, followed by pit latrines (18%), and open defaecation (16%). In the study area, differences were observed in the information gathered from the household questionnaires and the sanitary inspections at the water sources. About 2.3% of homes reported open defaecation, and 16% of sanitary inspections found open defaecation close to a water source. According to the WHO^6^, a licensed professional must travel to the site of the water supply as part of a sanitary inspection and thoroughly inspect the neighbourhood for circumstances that could lead to contamination. With that said, therefore, this study also recommends sanitary inspections to be done in water source locations, and this will require inspectors or community members to visit the fields and do observations according to checklists recording all hazards observed.

Sanitary risk scores were computed in accordance with WHO guidelines^6^. The present findings showed that drilled boreholes had a low risk, protected springs had a medium risk, and surface water from rivers or dams had a low to moderate risk. Unprotected springs and hand-dug wells were found to have high to very high risks. These findings unmistakably show that protected springs and groundwater from drilled boreholes may be safer than other water sources. Similar results were obtained by Bindra^29^; in addition, people typically view these sources as being of considerably higher quality than more common sources like ponds and streams^29^. These findings demonstrate how important it is to safeguard water sources like springs in order to prevent water contamination.

It was found that the H_2_S strip test can accurately detect faecal contamination of drinking water. This strategy has proven to be a useful tool for monitoring water quality and rapid screening of a large number of water samples^37^. Studies have shown that the majority of rivers that flow through communities were highly contaminated when compared to rivers exposed to less human activity^35,38^. Similar results were also obtained in the present study area, where samples collected at the Tshivhulani sampling points on the Mutshindudi River which are situated far from any households had negative H_2_S test values indicating low risk. Conversely, positive H_2_S test results were recorded during both the dry and wet seasons for the samples collected at sampling points on the Mvudi River in Maniini and the Luvuvhu River in Dididi, which are in close proximity to households. This finding clearly shows that human activity has a greater impact on the degradation of river water quality. In rural areas, it is necessary to teach residents and household members on how to use the H_2_S strip test for water quality assessment.

The use of sanitary inspection combined with the H_2_S strip test could be very effective as screening tools for faecal contamination of water sources in rural communities with low resources. This study identified the human and animal activities that may lead to water contamination especially with faecal matter using sanitary inspections and also showed the effectiveness of the H_2_S strip test in the study area. These findings further showed a strong positive correlation (*r*) between these two methods. These results indicate that rural community members should be made aware of the affordable tools that are available to ensure the safety of their drinking water and should receive training in the use of these tools. Inconsistency in microbial water quality testing in VDM was reported with the worst-case scenario of testing once a year^26^. Hence, these tools can be used in local water treatment plants for regular and consistent monitoring of water quality; however, they should not be used as replacement tests for the other laboratory-based water quality tests.

## Conclusion

It is evident from the data presented in this paper that H_2_S-producing organisms are consistently associated with the sanitary risk in water sources. Combining a sanitary inspection with an H_2_S strip test in the identification of faecal contamination in various water sources can assist in detecting faecal pollution originating from humans and warm-blooded animals in springs, dams, boreholes, hand-dug wells, and rivers. Water quality assessment in rural areas could become more common and widespread due to the availability of affordable tools such as H_2_S paper strip testing and sanitary inspections to identify human and animal excrement and agricultural practices linked to water pollution. Knowledge of contamination risks will result in the prevention of waterborne infections and a reduction in the number of diarrhoeal deaths. Effective water and sanitation management depends on having a thorough understanding of the local water resources, as well as their limitations and dangers. Rural communities need to be made aware of the risks associated with contamination of water sources and drinking water and discussion forums should be set up. This study calls for disseminating knowledge and educating people in rural communities with limited resources on these cost-effective tools for water quality monitoring. Governmental organisations should also become engaged, provide alternatives, and assist the community members in taking ownership of the management of their drinking water resources.

## Methodology

### Study site

**Figure 4:**
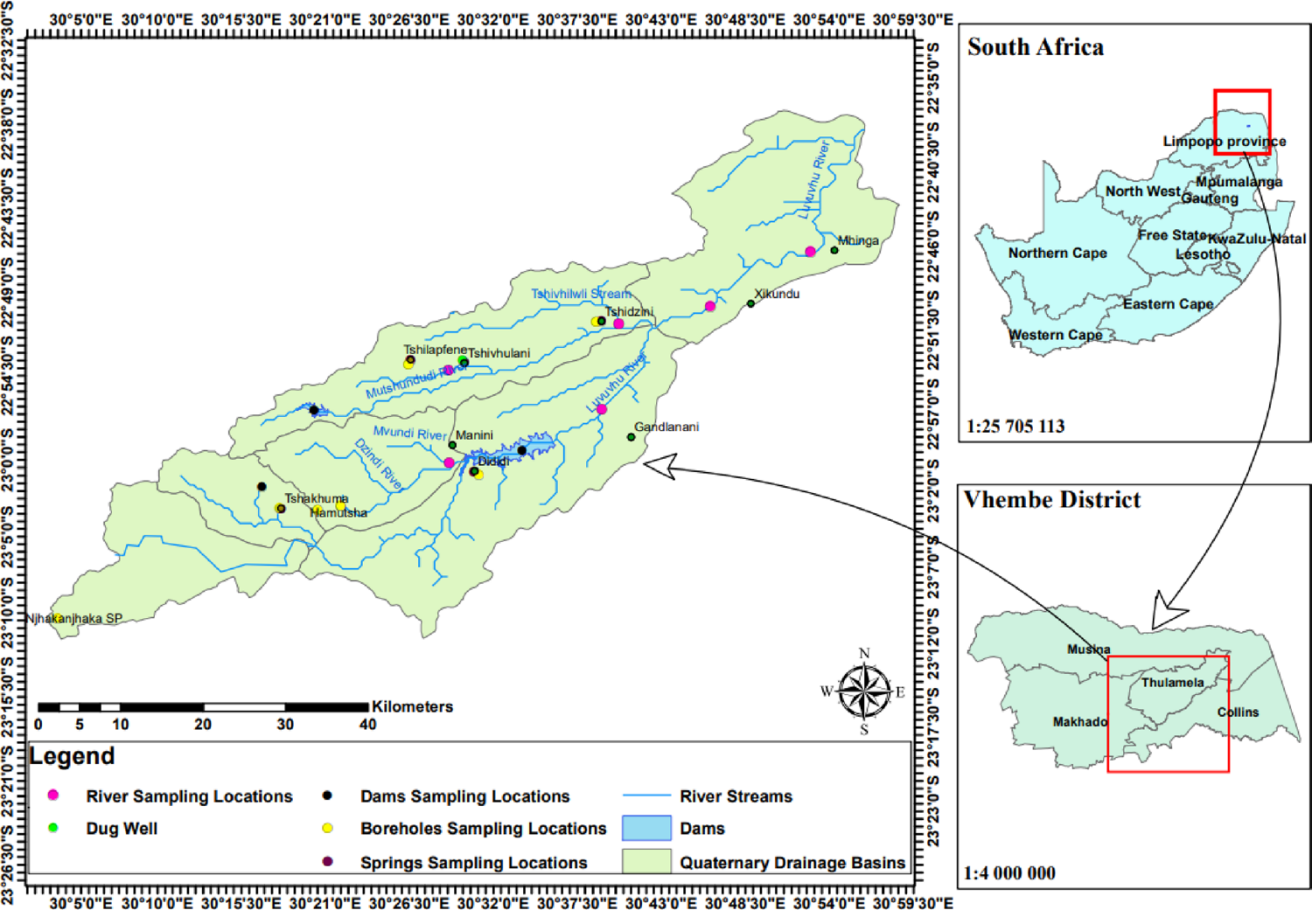
Map showing study area. Vhembe District Municipality is located in the northern part of Limpopo Province in South Africa.

This study was conducted in the Vhembe District Municipality of the Limpopo Province, South Africa (Fig. 4). The study population is estimated population of the Vhembe District Africa was approximately 1,393,949 people with 53.3% females and 46.7% males. This area has various water sources such as rivers, dams, springs, boreholes and hand-dug wells. It falls within the savannah biome with a sub-tropical climate that has hot, wet summers and cool winters. Vhembe District Municipality has a subtropical climate with distinct rainy and dry seasons. The average annual temperature in the area is around 22–24 °C. During the dry season, which typically occurs from April to September, the temperatures are cooler, with average highs ranging from 23 to 25 °C. Rainfall is highly seasonal, falling primarily during the summer months (October–March) with average highs ranging from 28 to 30 °C, and is heavily influenced by topography. The wettest months are January through March. The average annual precipitation ranges from 450 mm on the low-lying plains to more than 2 300 mm in the mountains. Subsistence farming supports a large proportion of the population.

**Figure 5:**
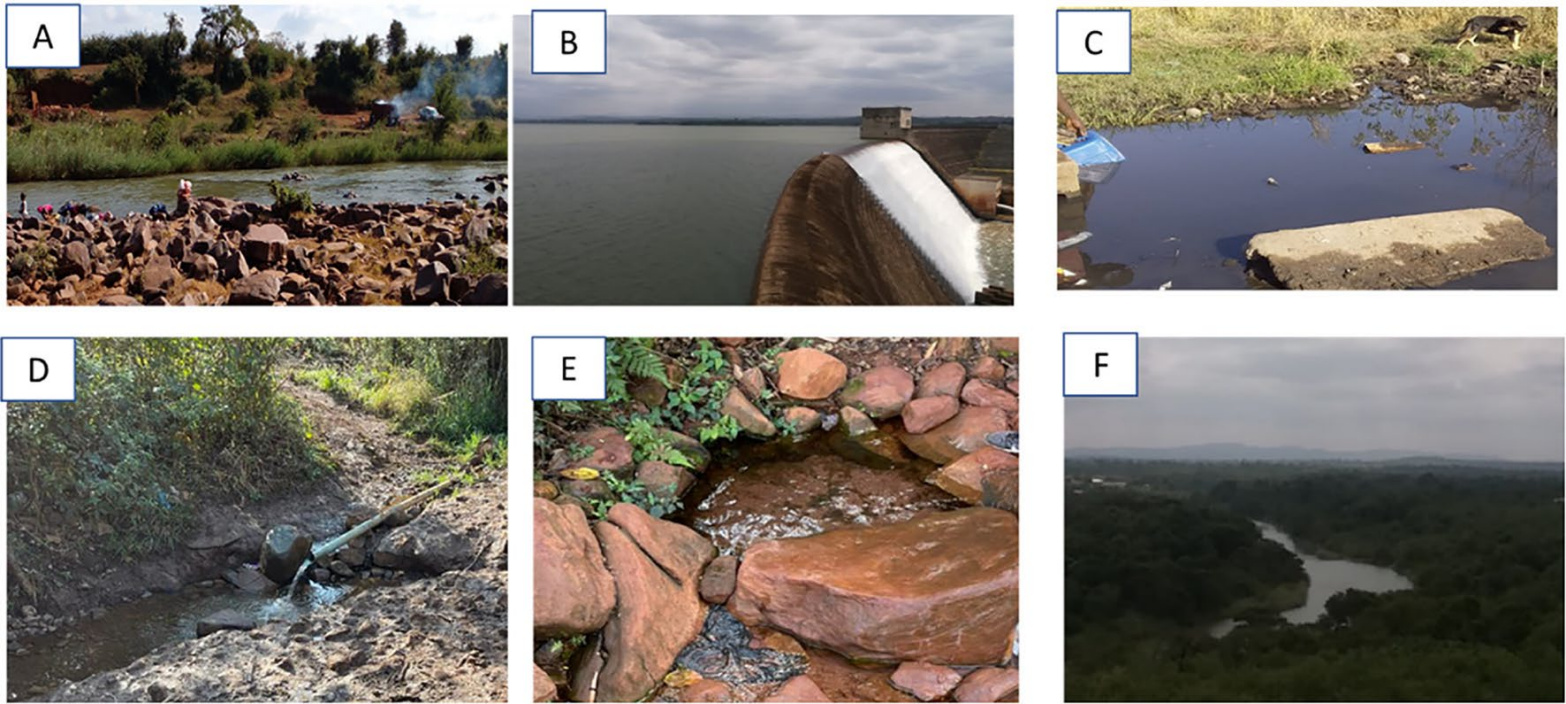
Some of the water sources in Vhembe District Municipality. (**A**) Luvuvhu River, Mhinga site; (**B**) Nandoni Dam; (**C**) Dididi Spring; (**D**) Tshivhulani Spring; (**E**) Tshilapfene Spring; (**F**) Luvuvhu River, Gandlanani site.

Forestry and agriculture are two of the most important land-use activities. This study concentrated on three rivers in the Vhembe District Municipality. They were chosen because of their proximity to human communities that rely on them for water for drinking, cooking, bathing and washing of clothes. The Luvuvhu River passes through three local municipalities of the Vhembe District Municipality, including Makhado, Thulamela and Collins Chabane. Nandoni Dam is the major dam in the Luvuvhu River catchment. Some of the water sources in the Vhembe District Municipality are indicated in Fig. 5.

### Ethical consideration

Ethical clearance approval was granted by the Faculty of Science Research Ethics Committee (FCRE) at the Tshwane University of Technology (TUT) and the Vhembe District Municipality. All research methods were performed in accordance with the relevant guidelines and regulations. Informed consent to participate in the study was obtained from the borehole owners in selected villages. The aim and objectives of the study were provided to the study participants and sampling permission was granted.

The Tshwane University of Technology Research Ethics Committee (FCRE 2019/08/003 (FCPS 03) (SCI) and 20 March 2020) gave their approval for the project.

### Collection of demographic information and water and sanitation data

Data for water sources and sanitation facilities were collected from Vhembe District Municipality in order to gain a general overview of the research area. Additionally, a general discussion about water and sanitation was held with municipal officials, local leaders, and community members. Briefly, a total of 35 rural villages in the Vhembe District Municipality were selected randomly from three different local municipalities. Water samples, demographic information (education, employment and diarrhoeal disease) and sanitation data (alternative water sources, purpose of water, water treatment method, and sanitation facility data) were collected between March 2020 and March 2021. Data from this preliminary inspection have been published in 2022 by Murei et al.^26^ For the purpose of the present study, these data were used to assess the sanitary risk. It was noted in this preliminary study that community members of the Vhembe District Municipality reportedly lack the necessary information and the understanding of the external risk linked to water resources and sanitation. Therefore, by utilising sanitary inspections, an evaluation of the water resources available in that area as well as of the hazards related to those resources was conducted.

### Sanitary inspection

A sanitary inspection was conducted to locate any risks and hazardous events that could affect the water resources. As part of the sanitary inspection, the location of each water source was visited and the local environment was thoroughly inspected for scenarios that could lead to contamination. A standardised questionnaire with a few predetermined questions was used to conduct sanitary inspections. Local languages were used for those who have difficulty in speaking or understanding English adequately. The most fundamental and common problems that could cause water system pollution were included in these surveys. Sanitary inspections were performed using a mixture of on-site inspection data and interviews of community members and water and wastewater operators. In general, these questions were written in a way that only YES or NO could be used as a response (Table 5). A risk factor is present when the response is YES, but it is absent when the response is NO. The level of safety of the water supply was then graded using a risk score (e.g., very high risk (7–8), high risk (5–6), medium risk (3–4) and low risk (1–2), which was determined by counting the number of YES responses as described by WHO^6^.

**Table 5:**
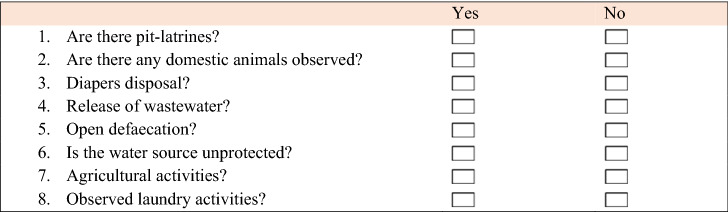
Sanitary inspection form.

### Sample collection

In the three local municipalities of the Vhembe District Municipality that were selected, 816 water samples were collected between March and April 2021 for the wet season and between June and August 2021 for the dry season. The rivers, dams, springs, boreholes, and hand-dug wells were all used to collect samples. For each season, samples were collected in four-cycle intervals at each sampling point, resulting in a total of 408 water samples (wet and dry). Table 6 shows the total number of samples collected for each sampling site per water source. The water sampling points were in different areas which included Mutshindudi River: Tshivhulani area, Ngwedi River: Tshidzini area, and Luvuvhu River: Mhinga area, Gandlanani area, and Dididi/Maniini area. Dams sampled included Nandoni Dam, Thathe Vondo Dam and Tshakhuma Dam. The springs located in Tshilapfene, Tshidzini, Dididi, Tshivhulani and Tshakhuma were sampled. A total volume of 1 L was collected using sterilised containers for microbiological water quality testing. Water samples were transported to the Microbiology Laboratory at Tshwane University of Technology in cooler boxes containing ice at 4 °C. The analysis was done within 6 h of collection.

**Table 6:** Samples collected in wet and dry season.

Sampling point	Thulamela LM		Collins Chabane LM		Makhado LM		Total
	Wet	Dry	Wet	Dry	Wet	Dry	
Catchment							
R	52	52	32	32	0	0	168
D/W	16	16	12	12	4	4	64
S	8	8	8	8	8	8	48
B/D	84	84	4	4	180	180	536
	160	160	56	56	192	192	816

*LM* local municipality, *R* river, *D/W* dam or weir, *S* spring, *B/D* borehole or hand-dug well.

### Water quality analysis using hydrogen sulphide strip test

The hydrogen sulphide strip test was performed using H_2_S paper strips (Macherey–Nagel, Monitoring & Control Laboratories, Johannesburg, South Africa), according to the manufacturer’s instructions with slight modifications. Briefly, a test tube containing approximately 9 mL of tryptic soy broth (Thermo Fisher Scientific, Johannesburg, South Africa) was prepared and 1 mL of water sample was added; thereafter the H_2_S paper strip was inserted into the test tube and secured by a ball of cotton wool so as to maintain it at the top centre of the tube. Results of the H_2_S strip test are reported as positive or negative. The colour changes of the paper strip from white to black indicated the presence of H_2_S gas, thereby indicating that water is contaminated with bacteria of faecal origin such as coliform bacteria.

### Statistical analysis

Microsoft Excel 2019 and Statistical Package for the Social Sciences (SPSS) Version 28 were used for statistical analysis. The correlation between concentration of sanitary score and H_2_S gas production in various water sources was analysed using Pearson’s correlation coefficient (r).

## Data Availability

The datasets used and/or analysed during the current study are available from the corresponding author upon reasonable request.

## References

[1] UNICEF, WHO (2021). Progress on Household Drinking Water, Sanitation and Hygiene (2000–2020) Who/Unicef Joint Monitoring Programme for Water Supply, Sanitation and Hygiene.

[2] WHO (2011). Water quality for drinking: WHO guidelines. Encycl. Earth Sci. Ser..

[3] Momba MNB, Tyafa Z, Makala N, Brouckaert BM, Obi CL (2006). Safe drinking water still a dream in rural areas of South Africa. Case study: The Eastern Cape Province. Water SA.

[4] Khabo-Mmekoa C, Momba M (2022). The burden of poor household drinking-water quality on HIV/AIDS infected individuals in rural communities of Ugu District Municipality, Kwazulu-Natal Province, South Africa. J. Biotechnol. Biomed..

[5] Matwewe F, Hyland K, Thomas J (2018). Locally produced hydrogen sulphide detecting water quality test kits increase household level monitoring in rural Tanzania. J. Water Health.

[6] WHO (2016). Quantitative Microbial Risk Assessment: Application for Water Safety Management.

[7] Luby SP (2008). Tubewell water quality and predictors of contamination in three flood-prone areas in Bangladesh. J. Appl. Microbiol..

[8] Rajgire AV (2013). Open defecation: A prominent source of pollution in drinking water in villages. Int. J. Life Sci. Biotechnol. Pharma Res..

[9] Abbasi A, Tahir SA, Asghar SA, Huang H (2022). Acute diarrheal outbreak in 2022 Karachi, Pakistan: To determine its clinical spectrum. Risk Fact. Compl..

[10] Al-Gasseer, N., El Bushra, H.E. & Yeneabat, A. Countrywide outbreak of acute watery diarrhea in Sudan, 2016–2018. (2019).

[11] Prüss-Ustün A (2019). Burden of disease from inadequate water, sanitation and hygiene for selected adverse health outcomes: An updated analysis with a focus on low- and middle-income countries. Int. J. Hyg. Environ. Health.

[12] Monyai M, Makhado RA, Novhe NO (2016). Water quality of the Luvuvhu River and its tributaries within the Thulamela Local Municipality, Limpopo Province, South Africa. Afr. J. Sci. Technol. Innov. Dev..

[13] Bansal N (2018). Industrial development and challenges of water pollution in coastal areas: The case of Surat, India. IOP Conf. Ser. Earth Environ. Sci..

[14] Gossweiler B, Wesström I, Messing I, Villazón M, Joel A (2021). Impact of land use change on non-point source pollution in a semi-arid catchment under rapid urbanisation in Bolivia. Water.

[15] Sun B (2012). Agricultural non-point source pollution in China: Causes and mitigation measures. Ambio.

[16] Ballesté E, García-Aljaro C, Blanch AR (2018). Assessment of the decay rates of microbial source tracking molecular markers and faecal indicator bacteria from different sources. J. Appl. Microbiol..

[17] Graham JP, Polizzotto ML (2013). Pit latrines and their impacts on groundwater quality: A systematic review. Environ. Health Perspect..

[18] Arefin MA, Mallik A (2017). Sources and causes of water pollution in Bangladesh: A technical overview. Bibechana.

[19] Wen X (2020). Microbial indicators and their use for monitoring drinkingwater quality-A review. Sustain..

[20] Yang H (2013). Accuracy of the H2S test: A systematic review of the influence of bacterial density and sample volume. J. Water Health.

[21] Manja KS, Maurya MS, Rao KM (1982). A simple field test for the detection of faecal pollution in drinking water. Bull. World Health Organ..

[22] Shingles, K. & Saltori, R. Community use of H2S (hydrogen sulphide) as a verification tool for water safety plans. in *Access to Sanitation and Safe Water: Global Partnerships and Local Actions: Proceedings of the 33rd WEDC International Conference*, 435–438 (2008).

[23] Wright JA (2012). The H2S test versus standard indicator bacteria tests for faecal contamination of water: Systematic review and meta-analysis. Trop. Med. Int. Heal..

[24] Khush RS (2013). H2S as an indicator of water supply vulnerability and health risk in low-resource settings: A prospective cohort study. Am. J. Trop. Med. Hyg..

[25] UNICEF (2007). Drinking-water quality monitoring and surveillance. Waterlines.

[26] Murei A (2022). Barriers to water and sanitation safety plans in rural areas of Limpopo Province. Water.

[27] WHO. *Guidelines for Drinking Water*. *Revision*, vol. 21 (2022).

[28] South Africa Constitution. *The Constitution of the Republic of South Africa, 1996 : As Adopted on 8 May 1996 and Amended on 11 October 1996*. *Republic of South Africa* (1996).

[29] Bindra D, Ravindra K, Chanana N, Mor S (2021). Assessment of on-site sanitation practices and contamination of groundwater in rural areas of Fatehgarh Sahib, Punjab, India. Environ. Dev. Sustain..

[30] Amatobi DA, Agunwamba JC (2022). Improved quantitative microbial risk assessment (QMRA) for drinking water sources in developing countries. Appl. Water Sci..

[31] Xu H, Gao Q, Yuan B (2022). Analysis and identification of pollution sources of comprehensive river water quality: Evidence from two river basins in China. Ecol. Indic..

[32] Fouche, P. S. O., Vlok, W. & Roos, J. C. *Establishing the Fishery Potential of Lake Nandoni in the Luvuvhu River, Limpopo Province*. (2013). http://www.wrc.org.za/KnowledgeHubDocuments/ResearchReports/1925-1-12.pdf.

[33] Islam MS (2016). Safe distances between groundwater-based water wells and pit latrines at different hydrogeological conditions in the Ganges Atrai floodplains of Bangladesh. J. Health. Popul. Nutr..

[34] Edokpayi JN, Enitan AM, Mutileni N, Odiyo JO (2018). Evaluation of water quality and human risk assessment due to heavy metals in groundwater around Muledane area of Vhembe District, Limpopo Province, South Africa. Chem. Cent. J..

[35] Traoré AN (2016). The impact of human activities on microbial quality of rivers in the Vhembe District, South Africa. Int. J. Environ. Res. Public Health.

[36] Ntanganedzeni B, Nobert J (2021). Flood risk assessment in Luvuvhu river, Limpopo province, South Africa. Phys. Chem. Earth.

[37] Pathak SP, Gopal K (2005). Efficiency of modified H2S test for detection of faecal contamination in water. Environ. Monit. Assess..

[38] Howard JJ, Cuffey K (2003). Freshwater mussels in a California North Coast Range river: Occurrence, distribution, and controls author (s): Jeanette K. Howard and Kurt M. Cuffey Published by: The University of Chicago Press on behalf of the Society for Freshwater Science Stab. J. N. Am. Benthol. Soc..

